# Production of flavorful alcohols from woods and possible applications for wood brews and liquors†

**DOI:** 10.1039/d0ra06807a

**Published:** 2020-11-01

**Authors:** Yuichiro Otsuka, Masanobu Nojiri, Norihisa Kusumoto, Ronald R. Navarro, Koh Hashida, Naoyuki Matsui

**Affiliations:** Department of Forest Resource Chemistry, Forestry and Forest Products Research Institute 305-8687 Tsukuba Ibaraki Japan yotuka@ffpri.affrc.go.jp

## Abstract

This work explores the utilization of wood for high-value production of novel alcoholic brews and liquors with natural flavors. The process capitalizes on our original wet-type bead milling (WBM) technology that enables direct enzymatic saccharification and alcohol fermentation of wood without chemical and heat treatment, resulting in the absence of toxic compounds. When alcohol-based products from various wood species, including *Cryptomeria japonica* (cedar), *Cerasus* × *yedoensis* (cherry), and *Betula platyphylla* (birch), were analyzed by SPME-GC-MS, different natural flavor components were found in each. Correlation analysis using Heracles NEO and ASTREE V5 showed that the alcohols from wood have different flavor and taste characteristics when compared with those of existing commercial liquors. From pilot-scale experiments, the yield of alcoholic brew per biomass amount was determined. Pilot-scale runs established the importance of optimum wood particle size during WBM for efficient alcohol production. Although the alcohol produced from wood must first be established as safe for human consumption, this is the first description of drinking alcohols produced from wood. This work may open up important avenues for the exploitation of wood resources toward food production to further advance the current state of forestry.

## Abstract 2

This work explores the utilization of wood for high-value production of novel alcoholic brews and liquors with natural flavors.

## Graphical abstract

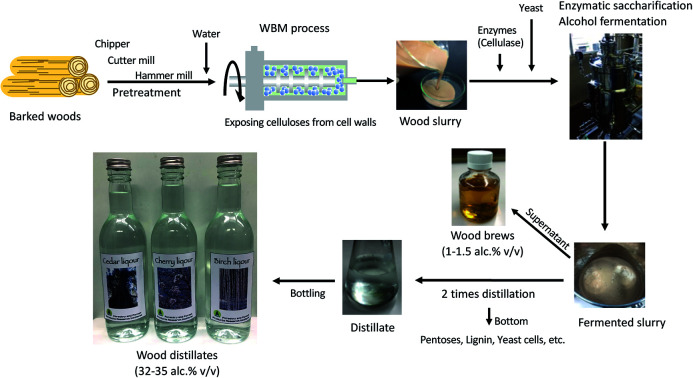

## Introduction

Used since ancient times as a raw material for construction, woodwork, pulp, and paper, wood is an important biomass. In recent years, sustainable green energy technologies have been developed for producing alcohols from wood for fuels.^1^ However, because the process of producing alcohol from wood involves chemical and heat treatment, it is difficult to scale up, given its environmental impact and high cost.^2^

We have developed a wet-type bead milling (WBM) technology that applies commercial bead mills used in colored ink production and precious metal mining as a pretreatment technique for wood. The WBM process allows direct enzymatic saccharification by grinding the wood in water to efficiently expose the cellulose and hemicellulose packed in the cell walls of the wood without heat or chemical treatment. Furthermore, we have developed a simultaneous enzymatic saccharification and comminution (SESC) technology that combines the WBM process with enzymatic saccharification by including cellulase and hemi-cellulase in the WBM process for industrial applications.^3,4^ We have previously reported that SESC technology can be used to obtain a sugar solution and unmodified lignin (SESC-lignin) as residue from wood.^4^ In addition, we have shown that SESC-lignin can be used as a functional material for various purposes.^5–8^ By combining the SESC process and anaerobic digestion, we are also developing technology to produce biogas by direct methane fermentation of wood.^9^ In this study, we sought to determine whether the combination of the WBM process, enzymatic saccharification, and a fermentation process might allow us to produce fermented food or drinks from wood because there is no chemical and heat process to produce toxic compounds as by-products.^10^

Although wood is not directly eaten, it is closely related to our food culture, and foods sometimes contain wood components. Many meal tools are made of wood, and wood barrels have been used to store foods and alcohols.

**Fig. 1:**
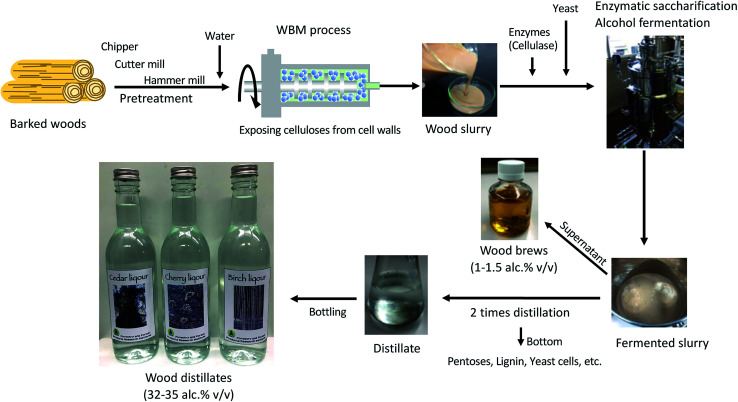
Schematic flowchart of the combined wet-type bead milling process, enzymatic saccharification, alcohol fermentation, and distillation process for food-grade processing of wood to produce wood brews and wood distillates.

The bead mill machine used for the WBM process, which can be used with water and wood alone, is also used for food processing, such as in the production of smooth chocolate cream. In addition, food-grade cellulase/hemi-cellulase enzymes are used as food additives to decrease the viscosity of vegetables, fruits juices, and other items.^11,12^ As the WBM process and enzymatic saccharification of wood can be performed with food-grade materials, we considered that novel alcohols for drinking could be produced from wood by combining WBM, enzymatic saccharification, and alcohol fermentation with yeast for brewing (Fig. 1).

The history of alcohol brewing is very long. For example, Li Leu *et al.* found evidence of 13 000 year-old traces of cereal-based beer brewing.^13^ Throughout this history, people have produced various brewed alcohols, such as wines, beers, Japanese sakes, and distilled liquors, such as vodka, whiskeys, and brandies. While the alcohols that we consume daily are made from the starch of cereals or sugars of fruits, we have no experience of consuming alcohols produced from wood celluloses. Therefore, we believe that this research is the world's first trial for producing alcohols from woods for drinking.

## Experimental section

### Materials

*Cryptomeria japonica* (Japanese cedar) wood was harvested at the Chiyoda nursery farm of Forestry and Forest Products Research Institute (FFPRI) in Kasumigaura City, Ibaraki, Japan, for this study. *Betula platyphylla* (Japanese white birch) wood was harvested from a forest used for research at Kyusyu University in Hokkaido, Japan. *Cerasus × yedoensis* (Yoshino cherry) wood was harvested at the center of FFPRI in Tsukuba City, Ibaraki, Japan. We purchased spring water from Mount Fuji at a supermarket for this study. The food-grade enzyme mixture of cellulase and hemi-cellulase, GODO-TCF (100 FPU per g), was purchased from Godo-syusei Co. Ltd., Tokyo, Japan. The yeast used for alcohol fermentation, the 901-gou, usually used for brewing Japanese sake made from rice, was purchased from Nihon Jyozo-kyokai, Tokyo, Japan. Samples of alcoholic drinks shown in Table 2 were purchased in a liquor store in Japan.

### Preparation of wood powder

Harvested woods were dried in air for 2 months. Then, surface cleaning of the dried woods using an electric plane was followed by bark removal. The resulting wood was chipped to a size of about 2 cm × 2 cm using a chipper (GSC283D, Ohashi Co., Ltd, Saga, Japan) and powdered to around 2 mm using a cutting mill (PULVERISETTE 15, Fritsch Japan Co., Ltd, Kanagawa, Japan). Wood powder of 2 mm was further processed using a hammer mill NH-30S (Sansyo industry Co., Ltd, Higashiosaka, Japan) with a 0.7 mm screen. The resulting wood powder passed through the 0.7 mm screen was used for pilot-scale bead mill treatment. For the laboratory-scale bead mill treatment, the wood powder was further processed by a vibratory sieve shaker (ANALYSETTE 3, Fritsch Japan, Co., Ltd, Kanagawa, Japan) with a 200 μm screen.

### Bead milling process

Laboratory-scale bead mill treatment was performed according to the following method. The LMZ015 (Ashizawa Finetech Co., Ltd, Chiba, Japan) was used as the bead mill. The beads were made of zirconia-reinforced alumina with a diameter of 0.5 mm. The filling rate of beads in the vessel was 80%. Spring water weighing 450 g was added to the bead mill tank, the water was circulated at a flow rate of 50 mL min^−1^ from tank to vessel, and the bead rotation speed was adjusted to 8 m s^−1^. Then, 50 g of wood powder of 0.2 mm was slowly added into the tank. After the injection was completed, the bead rotation speed was increased to 14 m s^−1^ and WBM processing was started. The processing time was 2 hours. After WBM processing was completed, the resulting wood slurry was collected in a beaker and sterilized at 105 °C for 30 minutes.

The bench plant-scale bead mill treatment was performed by the following method.

LME4 (Ashizawa Finetech Co., Ltd, Chiba, Japan) was used as the bead mill. Beads made of zirconia-reinforced alumina with a diameter of 2 mm were used. The filling rate of beads in the vessel was 80%. Spring water weighing 18 kg was added to the bead mill tank, and the water was circulated at a flow rate of 2 L min^−1^. The bead rotation speed was adjusted to 8 m s^−1^. 2 kg of 0.7 mm wood powder was slowly added into the tank. After injection was completed, the bead rotation speed was gradually increased, and when the speed reached 14.5 m s^−1^, WBM processing was started. The processing time was 6 hours. After the treatment was completed, the resulting wood slurry was collected in a stainless steel container and sterilized at 105 °C for 90 minutes.

### Enzymatic saccharification and alcohol fermentation of wood slurry

The laboratory-scale experimental procedures are as follows. The sterilized wood slurry was put into a 1 L jar fermenter and kept at 50 °C with stirring. 10 mL of enzyme solution GODO-TCF was added, for a concentration of 0.2 mL g^−1^ wood (corresponding to 40 FPU per g glucan), to the wood slurry, and enzymatic saccharification was performed for 24 hours. The 901-gou yeast cells, precultured in 25 mL of YPD medium for 24 hours, were collected by centrifugation. The resulting yeast pellet was resuspended in 5 mL of sterilized water to prepare a yeast suspension. After 24 hours of enzymatic saccharification, the temperature was equilibrated to 30 °C, and the yeast suspension was added to start alcohol fermentation. The sugars and alcohol were analyzed by HPLC every 24 hours, and fermentation was completed when glucose was exhausted and the amount of alcohol became constant.

The pilot plant-scale experimental procedures are as follows. 20 kg of the sterilized wood slurry was put into a 30 L stainless steel tank and warmed at 50 °C with stirring. 400 mL of enzyme solution GODO-TCF was added at a concentration of 0.2 mL g^−1^ wood (corresponding to 40 FPU per g glucan) to the wood slurry and enzymatic saccharification was performed for 24 hours. The 901-gou yeast cells, precultured in 1 L of YPD medium for 24 hours, were collected by centrifugation. The resulting yeast pellet was resuspended in 100 mL of sterilized water to prepare a yeast suspension. After 24 hours of enzymatic saccharification, the temperature was equilibrated to 30 °C, and the yeast suspension was added to start alcohol fermentation. The sugars and alcohol were analyzed by HPLC every 24 hours, and fermentation was completed when glucose was exhausted and the amount of alcohol became constant.

### Solid–liquid separation and distillation of alcohol fermented slurry

Solid–liquid separation of the alcohol-fermented slurry was performed by centrifugation at 9000 rpm × 10 min. The obtained supernatant was sterilized with a 0.22 μm filter, stored in a light-shielding glass bottle at 4 °C, and then used as the fermentation solution.

Distillation was performed by the vacuum distillation method as follows. 3.3 kg of the alcohol-fermented slurry was put into a 5 L round bottom flask and kept at 60 °C with stirring. Then, the entire distiller was depressurized to 150 hPa to start the distillation. Distillation was performed for 16.5 hours. The obtained distillation fraction was used as the primary distillate and stored in a light-shielding glass bottle at 4 °C. A part of the distillation residue was sampled for HPLC analysis.

The primary distillate was subjected to secondary distillation, as needed, which was carried out in the same manner that the primary distillation was. The obtained secondary distillation fraction was put in a light-shielding bottle and stored at 4 °C.

### Analytical procedures

The amount of remaining sugars and concentration of alcohol in the alcoholic fermentation slurry, the alcoholic fermentation liquid, and the distillate were analyzed by HPLC as described previously.^4^

Flavor component analysis of the alcoholic fermentation liquid and the distilled liquid was performed by SPME-GC-MS as follows. The alcohol concentration of all samples was adjusted to 10% (v/v) by adding ethanol or distilled water before SPME extraction. In a 7 mL pierce vial, 250 μL of each sample was placed with 100 mg NaCl and a magnetic stirrer. Each vial was screw-capped tightly with Mininert valves and equilibrated in a 50 °C aluminum block for 5 min before extraction. After equilibration, SPME fiber [50/30 μm divinylbenzene/carboxen/polydimethylsiloxane (DVB/CAR/PDMS) (Supelco Co., Bellefonte, PA, USA)] was placed into the headspace of the vial and extracted for 20 min at 50 °C with stirring. The SPME fibers were conditioned prior to use in a GC injector at 250 °C for 30 min.

After extraction, the SPME fiber was immediately injected into the SHIMADZU GCMS-QP2010 Ultra. Separation was achieved on a DB-WAX Ultra Inert column (30 m × 0.25 mm id, 0.25 μm film thickness; Agilent Technologies, Inc., Santa Clara, CA, USA). Helium was used as the carrier gas at a flow rate of 1.2 mL min^−1^ with a split ratio of 1 : 10, and the column temperature was programmed to hold at 40 °C (3 min hold) and then increase 10 °C min^−1^ to 240 °C (7 min hold). The injector temperature was 250 °C, and the detector temperature was 230 °C. Mass spectra were recorded over 40–300 amu range at 3.3 scan per s, with an ionization energy of 70 eV. The flavor components were identified by comparing mass spectra with those in the library data (NIST14 and FFNSC3).

### Comparative analysis of flavor components

A comprehensive analysis of flavor components was performed using the Heracles NEO system (Alpha MOS Japan Co., Ltd, Tokyo Japan) as follows. The alcohol concentration of all samples was adjusted to 5% (v/v) by adding ethanol or distilled water before analysis. In a 20 mL pierce vial, 3 g of each sample was placed and heated at 60 °C for 20 min with suspension. Then, 5 mL of headspace gas was sampled and injected into a two-dimensional gas chromatography system. MXT-5 (10 m, 180 μm ID, 0.4 μm) and MXT-WAX (10 m, 180 μm ID, 0.4 μm) were used as the GC column, hydrogen was used as the carrier gas with a split ratio of 1 : 10, and the column temperature was programmed to hold at 40 °C (10 s hold) and then increase 1.5 °C s^−1^ to 250 °C (60 s hold). The injector temperature was 220 °C, the detector temperature was 260 °C, and FID was used as the detector. A loading plot was created with the peak of the obtained two-dimensional chromatogram as a variable by AlfaSoft V15 software, and a correlation map was created on a two-dimensional plane by multivariate analysis.

### Comparative analysis of taste components

Analysis of taste components was performed using the ASTREE V5 system (Alpha MOS Japan Co., Ltd, Tokyo Japan) as follows. The alcohol concentration of all samples was adjusted to 5% (v/v) by adding ethanol or distilled water before analysis. In a 50 mL beaker, 25 mL of each sample was placed. Then, taste sensor arrays were put into the sample for 100–120 s until response signals become stable. Seven types of taste sensor arrays (AHS, CTS, NMS, PKS, CPS, ANS, and SCS) were used. Using the obtained taste signal data as a variable, a loading plot was created in AlfaSoft V15 software. The obtained loading plot was displayed as a correlation map on a two-dimensional plane by multivariate analysis.

## Results

### Composition of raw wood

*Cryptomeria japonica* (Japanese cedar), *Cerasus × yedoensis* (Yoshino cherry), and *Betula platyphylla* (Japanese white birch) wood were selected as raw materials for this study. In this paper, we refer to *Cryptomeria japonica, Cerasus × yedoensis*, and *Betula platyphylla* as cedar, cherry, and birch respectively.

Cedar, a softwood, had a lignin content of 32.59%, which was more than those of cherry, and birch, both hardwood. On the other hand, cedar had less holocellulose than cherry and birch. The content of α-cellulose was similar in these three wood species (Table S1†).

### WBM processing of woods

The particle sizes of cedar, cherry, and birch woods produced over the course of WBM processing using the LMZ015 laboratory-scale bead mill system were analyzed using a laser diffraction particle-size analyzer. The particle size of the softwood, cedar, decreased relatively quickly, reaching 5 μm or less within 1 hour and 2 μm or less within 90 minutes (Fig. S1†). After 120 min, the particle size reached submicron levels, at 0.67 μm. The hardwoods, cherry and birch, were 2.7 μm and 6.4 μm at 90 min and 1.9 μm and 4.1 μm at 120 min, respectively. Based on these results, the hardwoods, cherry and birch, were harder to grind than cedar, and birch was particularly difficult to grind. The resulting wood slurries were viscous, with pHs of around 4.2 (cedar), 4.6 (cherry), and 3.6 (birch).

### Enzymatic saccharification and alcohol fermentation of wood slurry

**Fig. 2:**
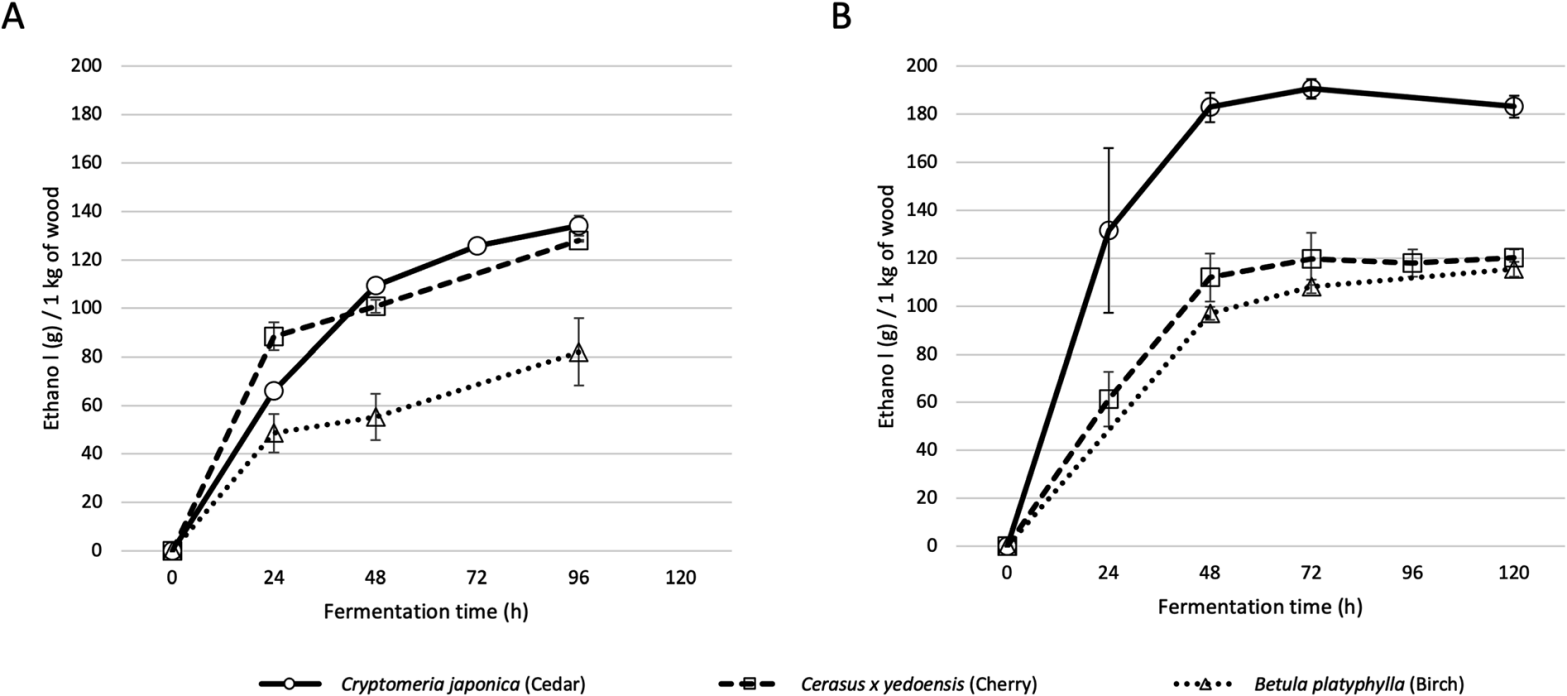
Time course of ethanol production by alcohol fermentation of wood slurries at laboratory-scale (A) and at bench plant-scale (B).

Fig. 2 shows the time course of ethanol production during alcohol fermentation. The enzymatic saccharification was carried out at 50 °C for 24 hours, with the addition of the enzyme. After 24 h, the temperature was set at 30 °C, and the yeast solution was added to start alcohol fermentation at 0 h of Fig. 2. For cedar, enzymatic saccharification released 262 g of glucose from 1 kg of wood and produced 134 g of ethanol. For cherry, 250 g of glucose was released from 1 kg of wood and 127 g of ethanol was produced. For birch, 168 g of glucose was released from 1 kg of wood and 82 g of ethanol produced.

### Concentration and recovery rate of alcohol by distillation and analysis of its flavor components

The alcohol concentrations of the wood alcohol fermentation slurries were around 1–2%. Because the alcohol concentration was low as it was for drinks, the distillation process was examined. The alcohol concentration and alcohol recovery rate for the cedar fermentation slurry were compared with those for the direct distillate and the distillate of only the supernatant after solid–liquid separation. The alcohol concentration in the cedar fermentation slurry was about 1.5%. When only the solid–liquid separated supernatant was distilled by single-batch distillation, the alcohol concentration was 5.8% (Fig. S2A†) and the alcohol recovery rate was 57.6% (Fig. S2B†). When the fermentation slurry was distilled directly, the alcohol concentration was 6.9% (Fig. S2A†) and the alcohol recovery rate was 77.5% (Fig. S2B†). Thus, direct distillation of the fermentation slurry produced a higher alcohol concentration and recovery rate. Because the alcohol concentration of the first distillate was as low as 5.8–6.9% when compared with existing distilled liquors (20–40%), a second distillate (>20% v/v) was obtained from an additional distillation of the first distillate and used in following experiments.

### Comparison of the flavor components of alcohols produced from cedar, cherry, and birch wood

Alcohol fermented slurries of cedar, cherry, and birch were distilled two times by single-batch distillation to obtain second distillates. The alcohol concentration of these distillates was adjusted at 10% v/v, and the flavor components were analyzed by SPME-GC-MS with DVB/CAR/PDMS fiber. Although many peaks were detected in the total ion-chromatogram, only the identified flavor components are shown in Table 1. In addition to ethanol, isobutyl alcohol (peak no. 1), isoamyl alcohol (peak no. 4), and phenethyl alcohol (peak no. 23) were detected in the three distillates. Many conifer-specific sesquiterpenes (peaks after 19 min) were only detected in the cedar distillate, which contained many cedar-specific flavor components. Sesquiterpenes were not detected in the hardwood distillates, cherry and birch. Instead, isoamyl acetate (peak no. 2), 1-hexanol (peak no. 7), 2-ethyl-1-hexanol (peak no. 11), 1-octanol (peak no. 14), acetophenone (peak no. 15), and 2-phenethyl acetate (peak no. 20) were distinctively detected in the cherry and birch distillates. In addition, 1-butanol, benzaldehyde, and benzyl alcohol were uniquely detected in the cherry distillate. 1-Pentanol, 6-methyl-5-hepten-2-one, 1-heptanol, 6-methyl-5-hepten-2-ol, 3-ethyl-1-hexanol, 2-pentyl-2-cyclopenten-1-one, geranylacetone, and gamma-nonalactone were only detected in the birch distillate, and the amount of phenethylacetate was higher than that in the cherry distillate. Chromatograms of SPME-GC-MS analysis of cedar, cherry, and birch are also shown in Fig. S3.†

**Table 1:** Flavor components identified in the SPME-GC-MS analysis of three wood distillates^a^

Peak no.	Compound name	Birch	Cherry	Cedar	Peak no.	Compound name	Birch	Cherry	Cedar
1	Isobutyl alcohol	✓*	✓*	✓	19	Cubenene	—	—	✓
2	Isoamyl acetate	✓	✓	Tr	20	2-Phenethyl acetate	✓*	✓	Tr
3	1-Butanol	—	✓	—	21	Geranylacetone	✓	—	—
4	Isoamyl alcohol	✓*	✓*	✓*	22	Benzyl alcohol	—	✓	—
5	1-Pentanol	✓	—	—	23	2-Phenethyl alcohol	✓*	✓*	✓
6	6-Methyl-5-hepten-2-one	✓	—	Tr	24	Gleenol	—	—	✓
7	1-Hexanol	✓	✓	—	25	γ-Nonalactone	✓	—	—
8	Acetic acid	—	—	Tr	26	Cubenol	—	—	✓
9	1-Heptanol	✓	—	—	27	1-Epicubenol	—	—	✓*
10	6-Methyl-5-hepten-2-ol	✓	—	—	28	γ-Eudesmol	—	—	✓*
11	2-Ethyl-1-hexanol	✓	✓	Tr	29	τ-Muurolol	—	—	✓
12	3-Ethyl-4-methylpentan-1-ol	✓	—	—	30	α-Muurolol	—	—	✓*
13	Benzaldehyde	—	✓	—	31	α-Eudesmol	—	—	✓
14	1-Octanol	✓	✓	—	32	β-Eudesmol	—	—	✓*
15	Acetophenone	✓	✓	—	33	Neointermedeol	—	—	✓
16	α-Terpineol	—	—	Tr	34	Cryptomerione	—	—	✓
17	2-Pentyl-2-cyclopenten-1-one	✓	—	—	35	Juniper camphor	—	—	✓
18	δ-Cadinene	—	—	Tr					

a Asterisk: peaks detected more than 5% of total peak area. Trace (Tr): peaks detected less than 0.1% of total peak area. Hyphen: not detected.

### Correlation between existing alcoholic drinks and alcohols made from wood in flavor components and taste sensor signals

**Fig. 3:**
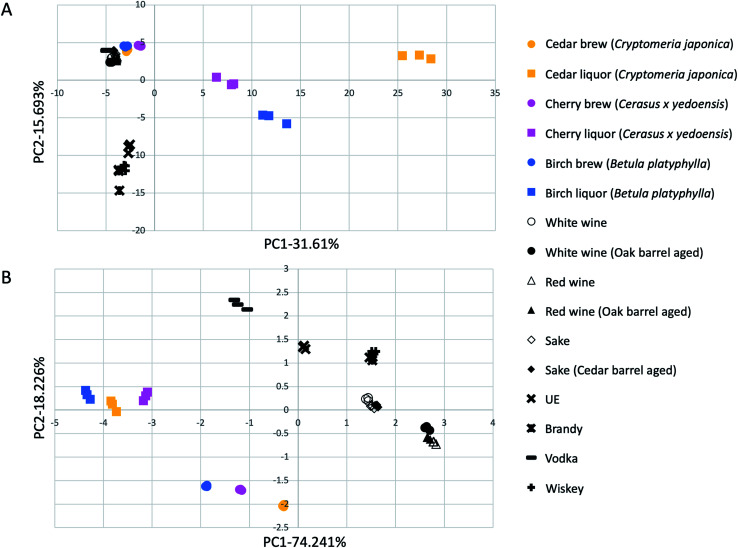
Correlation map of flavor components (A) and taste sensor signals (B) between existing alcoholic drinks and alcohols made from woods.

The flavor components detected in wood brews, wood distillates, and existing alcoholic drinks were compared by multivariate analysis following two-dimensional GC analysis using the Heracles NEO system (Alpha MOS Japan Co., Ltd, Tokyo, Japan). Table 2 shows information about the alcohol samples for the correlation analysis in this study. All alcohol samples were adjusted to have an alcohol concentration of 5% v/v and then analyzed by two-dimensional GC. Fig. 3A shows the results obtained by comprehensively comparing the peaks by multivariate analysis and displaying them on a two-dimensional map. All brewed alcohols and wood brews were closely clustered. Vodka also clustered near the brewed alcohols group even though vodka is a distilled alcohol. This may be because vodka is produced by the continuous distillation method, with its flavor components not concentrated. In the existing alcoholic drinks, brewed and distilled alcohols were positioned apart on the *y*-axis, but all of them were positioned between −5 and 0 on the *x*-axis. On the other hand, the wood distillates were positioned far apart along the *x*-axis (Fig. 3A).

**Table 2:** List of alcohol samples used for this study

Name of alcoholic samples	Alcohol type	Raw materials	Barrel aged	Specific name	The country of origin
Cedar brew (*Cryptomeria japonica*)	Brewed alcohol	Wood (*Cryptomeria japonica*)	None	This study	This study
Cedar liquor (*Cryptomeria japonica*)	Distilled alcohol	Wood (*Cryptomeria japonica*)	None	This study	This study
Cherry brew (*Cerasus × yedoensis*)	Brewed alcohol	Wood (*Cerasus × yedoensis*)	None	This study	This study
Cherry liquor (*Cerasus × yedoensis*)	Distilled alcohol	Wood (*Cerasus × yedoensis*)	None	This study	This study
Birch brew (*Betula platyphylla*)	Brewed alcohol	Wood (*Betula platyphylla*)	None	This study	This study
Birch liqour (*Betula platyphylla*)	Distilled alcohol	Wood (*Betula platyphylla*)	None	This study	This study
White wine	Brewed alcohol	Grape (Chardonnay)	None	Chablis La Pierrelee	France
White wine (oak aged)	Brewed alcohol	Grape (Chardonnay)	Yes	Chablis Cuvee Vieelles Vignes	France
Red wine	Brewed alcohol	Grape (Barbera)	None	Barbera D'Asti	Italy
Red wine (oak aged)	Brewed alcohol	Grape (Barbera)	Yes	Barbera D'Asti Superiore	Italy
Sake	Brewed alcohol	Rice (Hanafubuki)	None	Denshu	Japan
Sake (cedar barrel aged)	Brewed alcohol	Rice (Sasanishiki/Kuranohana)	Yes	Ichinokura Taruzake	Japan
UE	Distilled alcohol	Grape (Malvasia)	None	La Malvasia di NONINO UE	Italy
Brandy	Distilled alcohol	Grape (Ugni blanc)	Yes	Hennessy V.S.O.P	France
Vodka	Distilled alcohol	Barley	None	FINLANDIA	Finland
Whisky	Distilled alcohol	Barley	Yes	The MACALLAN 12 y old	Scotland

Next, the samples in Table 2 were also compared by taste sensor analysis using the ASTREE V5 system (Alpha MOS Japan Co., Ltd, Tokyo, Japan) and multivariate analysis. The fermented alcohols from wood and distillates of wood alcohols were located at distinct positions from any existing alcoholic drinks (Fig. 3B). Based on these results, the fermented alcohols from wood and wood distillates exhibit different taste characteristics from existing alcoholic drinks.

### Bead milling, enzymatic saccharification, and alcohol fermentation of wood on a pilot plant-scale

In order to obtain basic data for the social implementation of producing drinking alcohol from wood, we attempted alcohol production from wood at the bench plant-scale. Using LME4, which has a vessel volume about 25 times that of the LMZ015 laboratory-scale bead mill, the amount of processed wood powder was scaled up from 50 g to 2 kg. The results of alcohol fermentation are shown in Fig. 2B.

For cedar, 372.6 g of glucose was released from 1 kg of dry wood, and 190.5 g of ethanol was produced. For cherry wood, 267.9 g of glucose was released and 120 g of ethanol was produced. For birch wood, 233.9 g of glucose was released and 115.3 g of ethanol was produced. Unexpectedly, the amounts of glucose released and the amounts of ethanol produced were improved for all wood species when compared with the laboratory-scale procedure with LMZ015.

Next, the number of required distillations and the alcohol recovery rates were examined. The alcohol concentration of the initial fermented slurry was 1.0–1.8% v/v. When the fermented wood slurry was distilled by single-batch distillation, the alcohol concentration was 6.4–9.7% v/v. When this distillate was distilled again, the alcohol concentration was 31.5–35.0% v/v. When the amount of alcohol in the fermentation slurry was 100%, the recovery rate was 57.8–63.7% in the first distillation and was 34.8–49.3% in the second distillation. The alcohol remaining in the bottom fraction of the second distillation was 12.7–21.4%.

**Fig. 4:**
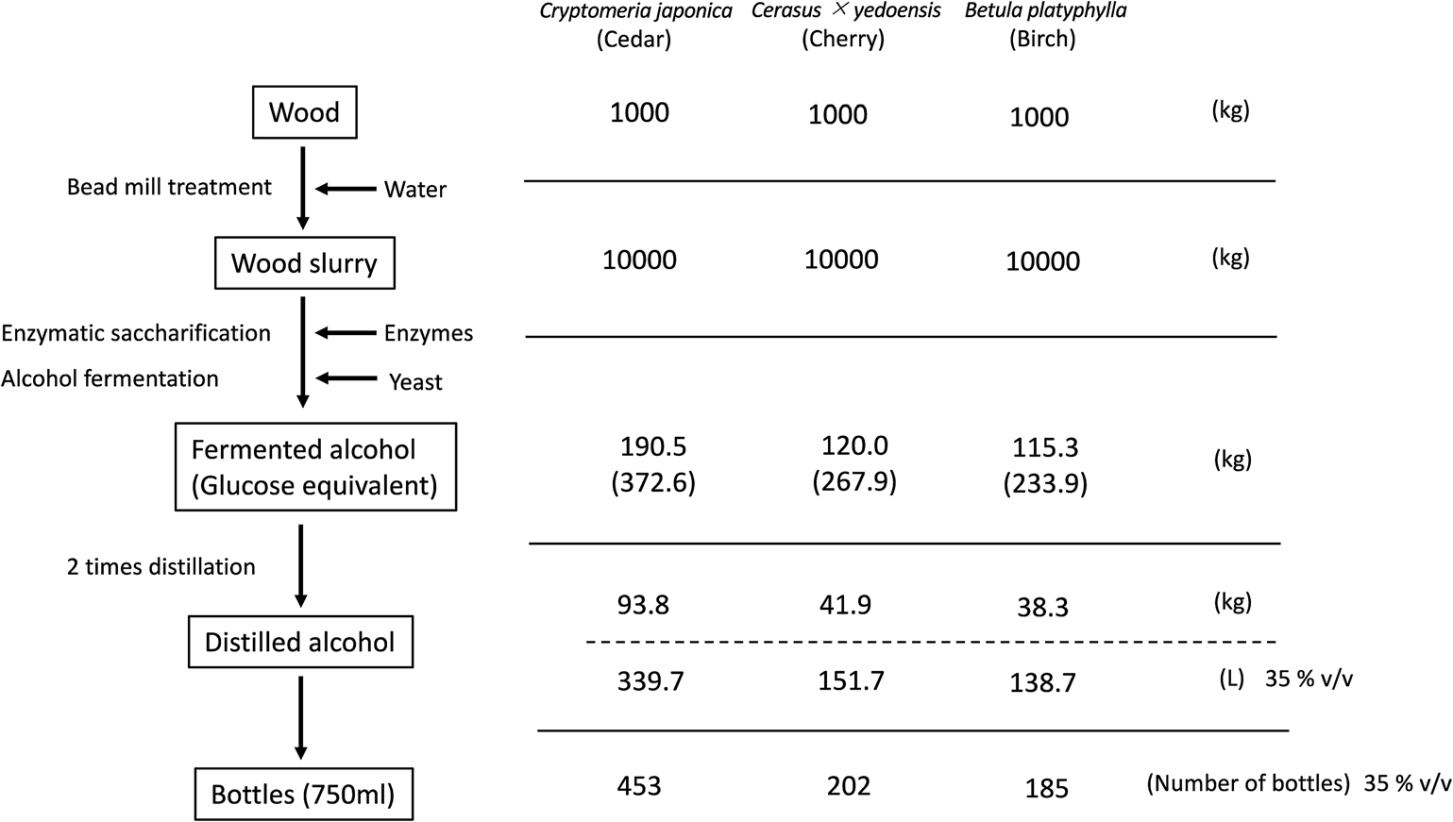
The yields of each process and calculated final production number of bottles with 35% v/v alcohol from each wood species.

Combining the above results from the pilot-scale experiments, Fig. 4 shows the mass balance for each process if alcohol were produced from 1000 kg of each wood. When cedar wood was used as a raw material, 372.6 kg of fermentable sugar would be produced by bead mill treatment and enzymatic saccharification, and 190.5 kg of ethanol would be produced. Using two rounds of distillation, 93.8 kg of ethanol would be recovered and 339.7 L of 35% v/v alcohol would be produced. This is equivalent to 453 bottles, each containing 750 mL. Similarly, for cherry wood, 41.9 kg of ethanol would be recovered using two rounds of distillation, and 151.7 L of 35% v/v alcohol would be produced. This is equivalent to 202 bottles for 750 mL. For birch wood, 38.3 kg of ethanol would be recovered using two rounds of distillation, and 138.7 L of 35% v/v alcohol would be produced. This is equivalent to 185 bottles for 750 mL.

## Discussion

The WBM process, which allows direct reaction between cell wall components and enzymes without chemical or thermal treatment, may be used to extend the application of lignocellulosic biomass to the food sector. In this study, we applied the WBM process to produce alcohols for drinking from the cedar, cherry, and birch wood species. These three wood species are closely related to food culture in Japan. Cedar barrels are used to store soy sauce, miso, and Japanese sake. Cherry is usually used for smoke chips. Birch is also used for disposable chopsticks, toothpicks, and ice cream sticks. Since foods containing components of cedar, cherry, or birch are eaten on a daily basis in Japan, these woods could be considered safe for human consumption.

Fig. 1 shows our proposed alcohol production process workflow. In this process, enzymatic saccharification and alcohol fermentation with yeast are carried out with whole woods after the WBM process. The pH of the wood slurry produced from the WBM process was maintained between 3.6 and 4.6 by dissolving acidic components from the wood. This pH condition was suitable for enzymatic saccharification and alcohol fermentation. Therefore, the addition of buffers and other chemicals was not necessary for the following processes. No significant inhibition was observed in the saccharification/fermentation process although the wood components contained a large amount of unfermented materials such as lignin. In the case of cedar wood, the maximum saccharification rate was 88.4% per alpha-cellulose, with an enzyme concentration of 0.2 mL g^−1^ wood biomass (corresponding to 40 FPU per g glucan above) (Fig. 2, Table S1†). On the other hand, established methods such as the SPORL process, which combines mild thermochemical treatment with enzymatic reaction, has been reported to achieve higher efficiency (90%) at lower enzyme loading (15 FPU).^14^ Although the fermentation process in this study required more enzyme than the SPORL process does, the enzymatic saccharification rate was similar.

The production of ethanol for fuel from lignocellulose using chemical treatment is a costly process, requiring pretreatment (milling processes), recovery of chemicals, and waste water treatment.^15^ Although milling is required as pretreatment in the process developed in this study, shown in Fig. 1, it does not require a separate chemical recovery step. In addition, the cost of wastewater treatment should be low, as no toxic reagents or environmental pollutants are involved in our method. Since the main power of the WBM process is rotational power, the driving cost can be reduced by using watermills and windmills.^4^ Bioethanol technology is being developed as a replacement for fossil fuels. According to data from the U.S. Bioenergy Statistics from the United States Department of Agriculture, the price of bioethanol from corn is estimated at $1.76 per gasoline equivalent gallon.^16^ This means the estimated value of ethanol for fuel is about $0.47 per gasoline equivalent liter. On the other hand, for drinking alcohol purposes, the average price of drinking alcohol in the U.S. is $3.36 per ounce ethanol,^17^ or $113.62 per L ethanol. This means that there is more than a 200-fold difference in the target price between fuel and beverage use even when tax rates are factored into this price. Therefore, we believe that the process in Fig. 1 should be profitable for producing alcohol from woods for drinking.

The results from the bench plant-scale show alcohol production efficiencies almost equal to, or better than, those at the laboratory-scale (Fig. 2). This result indicates that there is almost no problem in scaling up. Large-scale bead mill equipment, with vessel volumes of more than 100 times that of LME4, is already operated at color ink factories and precious metal mining sites. Since enzymatic saccharification, alcohol fermentation, and distillation processes require almost the same equipment as those used in the production of existing alcoholic drinks, it should not be difficult to scale up these processes for business.

Although many peaks were detected in the SPME-GC-MS analysis using DVB/CAR/PDMS fiber, only the identified components are shown in Table 1. The term “wood alcohol” is sometimes used to refer to methyl alcohol. However, methanol was not detected in any of the three distilled alcohols. Unique flavor components were detected in the distillate alcohols of cedar, cherry, and birch. The common components detected in all three distillates were isobutyl alcohol, isoamyl alcohol, and 2-phenetyl alcohol; these are known to evoke malty,^18^ flowery,^19^ and rose-like flavors,^18^ respectively.

Many sesquiterpene carbohydrates and alcohols were detected in the cedar distillate, in addition to the common components. The detected sesquiterpenes had almost the same composition as that of the essential oil extracted from used sake barrels made from Japanese cedar.^20^ In addition, only a small amount of aliphatic alcohols and other flavor components were detected in the cedar distillate. In fact, the cedar distillate had a specific cedar wood flavor, similar to cedar barrel-aged sakes.

Isoamyl acetate, 1-hexanol, 2-ethyl-1-hexanol, 1-octanol, acetophenone, and 2-phenetyl acetate were detected as common components in both cherry and birch distillates. These components were either undetectable or in trace amounts in cedar distillates. The aliphatic alcohols 1-hexanol, 2-ethyl-1-hexanol, and 1-octanol evoke freshly mown grass,^21^ aromatic,^22^ and penetrating aromatic odors,^23^ respectively. Isoamyl acetate, acetophenon, and 2-phenetyl acetate evoke banana,^24^ flowery,^25^ and fine rose and sweet honey flavor components,^26^ respectively. In addition to these common flavor components, specific flavor components such as benzaldehyde and benzyl alcohol, which are thought to be sugar metabolites of yeast,^27^ were detected only in the cherry distillate. Benzaldehyde produces a bitter almond flavor^27^ and benzyl alcohol produces jasmine flower flavor.^28^ In fact, the distillate of cherry had a sweet and flowery flavor and weaker wood flavor than the cedar distillate.

More aliphatic alcohols, geranylacetone, and gamma-nonalactone were specifically detected in birch distillate. Geranylacetone is a leafy flavor component,^29^ while the gamma-nonalactone contained in red wine^30^ and barrel-aged rum,^31^ produces an aged sweet flavor. The birch distillate had a weaker woody flavor than the cedar distillate, with more fruity, sweet, lush, and aged flavors. These results suggest that different flavored alcohols can be produced from different tree species.

To compare existing alcoholic drinks with the wood brews and distillates, multivariate analysis was used to compare the flavor components. All the existing alcoholic drinks converged in the range of −5 to 0 on the *x*-axis, while on the *y*-axis, existing brewed and distilled alcohols were largely separated. On the other hand, the wood distillates were located far away from existing alcoholic drinks along the *x*-axis. This result indicates that the wood distillates have a different flavor character than existing distilled alcohols. In addition, the cedar, cherry, and birch distillates were also located at a distance from one another. Therefore, alcohols produced from wood have different flavor characteristics, depending on the tree species. This result is consistent with the results of the analysis of flavor components by SPME-GC-MS.

A multivariate comparison of the results of the taste sensor analysis showed that the existing alcoholic drinks, wood brews group, and wood distillates group were differentially located (Fig. 3B). This indicates that wood brews and wood distillates have different characteristics from those of any existing alcoholic drinks in their taste components.

Interestingly, in both the flavor and taste multivariate analyses, the existing barrel-aged alcohols were close to the non-barrel-aged alcohols made from the same raw materials, and were located at a distance from the wood brews and wood distillates (Fig. 3). These results indicate that wood brews and wood distillates produced by direct saccharification and fermentation of wood cellulose have different flavor and taste characteristics from those of any existing alcohols that were flavored by soaking in wood.

Approximately 35% v/v alcohol could be produced by two rounds of distillation. This alcohol concentration is about the same as a typical liqueur and slightly lower than whiskey. Therefore, distilled alcohols made from wood have a sufficient alcohol concentration for distilled liquor.

For cedar, it was calculated that 453 bottles of distilled alcohol at 35% v/v could be produced in 750 mL bottles from one ton of raw material (Fig. 4). In Japan, a 30 cm (diameter) × 4 m (length) cedar log(about 113 kg dry weight) traded for about 3800 JPY (about $32) in 2018.^32^ Based on our calculations, about 50 bottles of distilled cedar alcohol can be made from one log, meaning the cost of cedar wood per bottle would be about $0.64.

Generally, the thickness of the cell walls of wood is 2–4 μm.^33^ The softwood, Japanese cedar, is processed to less than 2 μm by WBM treatment, which means that cellulose is efficiently exposed from the cell wall (Fig. S1†). However, the hardwoods, cherry and birch, could only be processed to 2–5 μm under the present conditions (Fig. S1†), suggesting that cellulose is not sufficiently exposed from the cell wall. Therefore, the final particle size of the wood slurry may affect the efficiency of enzymatic saccharification.

Low saccharification efficiency leads to low alcohol concentration in the fermented alcohol. This would lead to more losses in the following distillation process and cause a significant difference in final alcohol production. If suitable WBM process conditions were to be found for hardwood in the future, production efficiency from cherry and birch would be improved.

For the process shown in Fig. 1, chipper, cutter mill, hammer mill, and bead mill wood grinding was completed within 2–3 days; enzymatic saccharification and alcohol fermentation were completed in 5–7 days; and the distillation process is completed in 4 days. Consequently, all steps can be completed within 2 weeks. The process in Fig. 1 is basic and can be modified for various purposes. For example, the fermentation process can be set at low temperatures and extended to increase the flavor components produced by yeast and to maintain the natural flavor of the wood. It is also possible to select the flavor components to be concentrated by adjusting the pressure and temperature of the distillation process or to increase or decrease the alcohol concentration by changing the number of distillations. These conditions can be changed at will, depending on what kind of alcohol is intended to be produced and what kind of wood species is used as the raw material.

In human history, there have been no situations wherein people routinely drank alcohol made from wood. Therefore, it will be necessary to assess the human safety of these alcohols. Our laboratory is currently conducting a safety test of the alcohols produced from wood. In addition, a large amount of fermentation residue is generated in the process of producing alcohols from wood. The fermentation residue contains a large amount of lignin, which is one of the main components of wood. It is also necessary to study the utilization of this fermentation by-product in the future.

The production of different flavors depending on the wood species is expected to lead to the discovery of new value for currently unused tree species. Furthermore, other fermentation processes, such as acetic acid fermentation and lactic acid fermentation, may be applied to this process. Future research would perhaps allow us to develop technologies to make vinegar and lactic acid drinks from woods.

Finally, various types of alcoholic drinks throughout human history have fostered various food cultures. The discovery that alcohols with distinct flavors can be made from wood could perhaps be followed by the development of a new food culture.

## Conclusions

The wet-type bead milling (WBM) process enables direct saccharification and alcohol fermentation of wood without chemical or heat treatment. By using food-grade cellulase and yeast, we propose the possibility of producing potable brews and liquors from wood for the first time. The distilled alcohols made from cedar, cherry, and birch have distinct flavor characteristics. This suggests that every wood species can produce alcohol with a unique character. In addition, correlation analysis showed that the alcohols made from woods are also different from existing commercial alcoholic drinks. These results are expected to contribute not only to the development of the forestry industry but also to the creation of new food cultures.

## Conflicts of interest

There are no conflicts to declare.

## Supplementary Material

RA-010-D0RA06807A-s001
