# Supplementary material for: Production of flavorful alcohols from woods and possible applications for wood brews and liquors
Source: RSC Adv. 2020 Nov 1;10(65):39753–62. doi: 10.1039/d0ra06807a (PMC9057436; doi:10.1039/d0ra06807a)
Supplement: RA-010-D0RA06807A-s001 [file RA-010-D0RA06807A-s001.pdf]

Electronic Supplementary Material (ESI) for RSC Advances.
This journal is © The Royal Society of Chemistry 2020

Table S1 Components of Japanese cedar, Cherry and Birch wood

	Wood components (wt%)			
	Extractives	Klason Lignin	Holo-cellulose	alpha-cellulose
Cryptomeria japonica (Cedar)	3.59 ±0.03	32.59 ±0.06	65.20 ±2.00	44.07 ±1.08
Cerasus × yedoensis (Cherry)	3.14 ±0.09	23.43 ±1.04	74.01 ±1.46	43.18 ±0.96
Betula platyphylla (Birch)	2.52 ±0.07	20.84 ±0.14	75.50 ±0.70	45.95 ±1.09

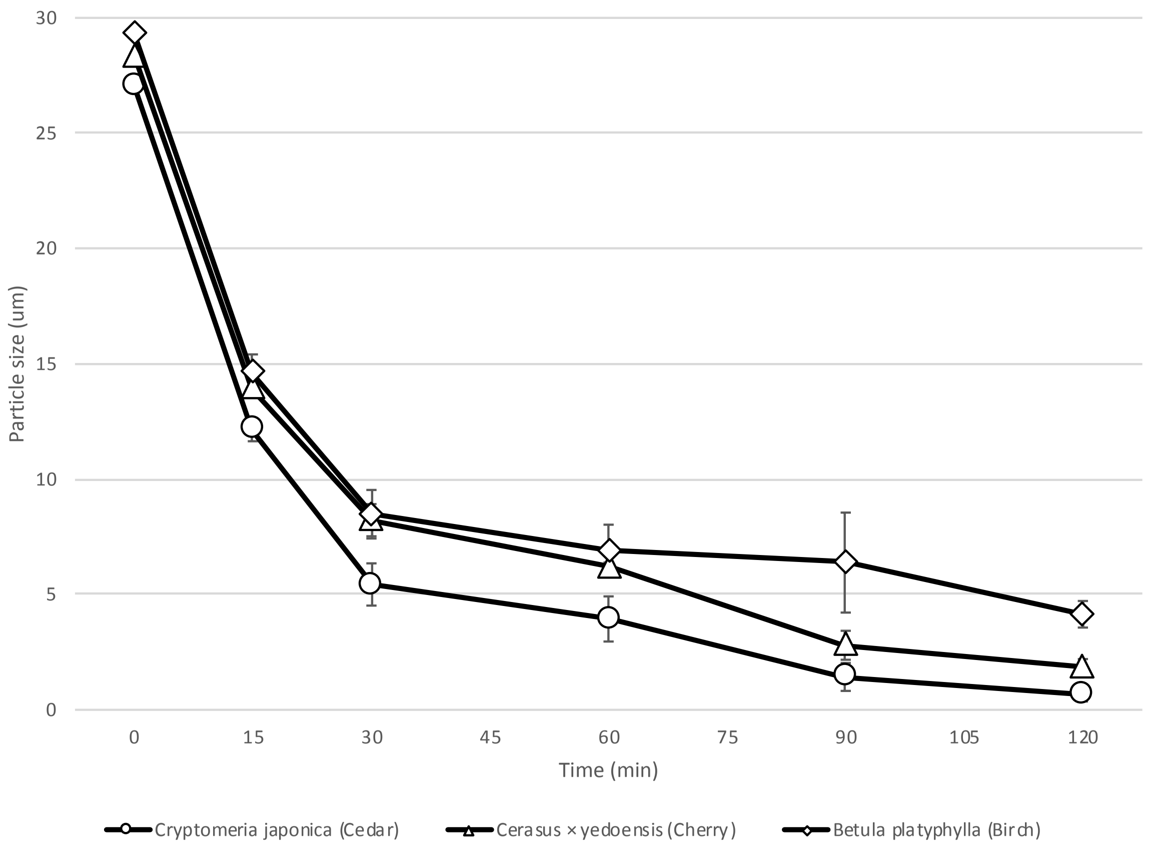

Fig.S1 Time course particle size of wood with bead mill treatment.

Figure S1. *Otsuka et al.*

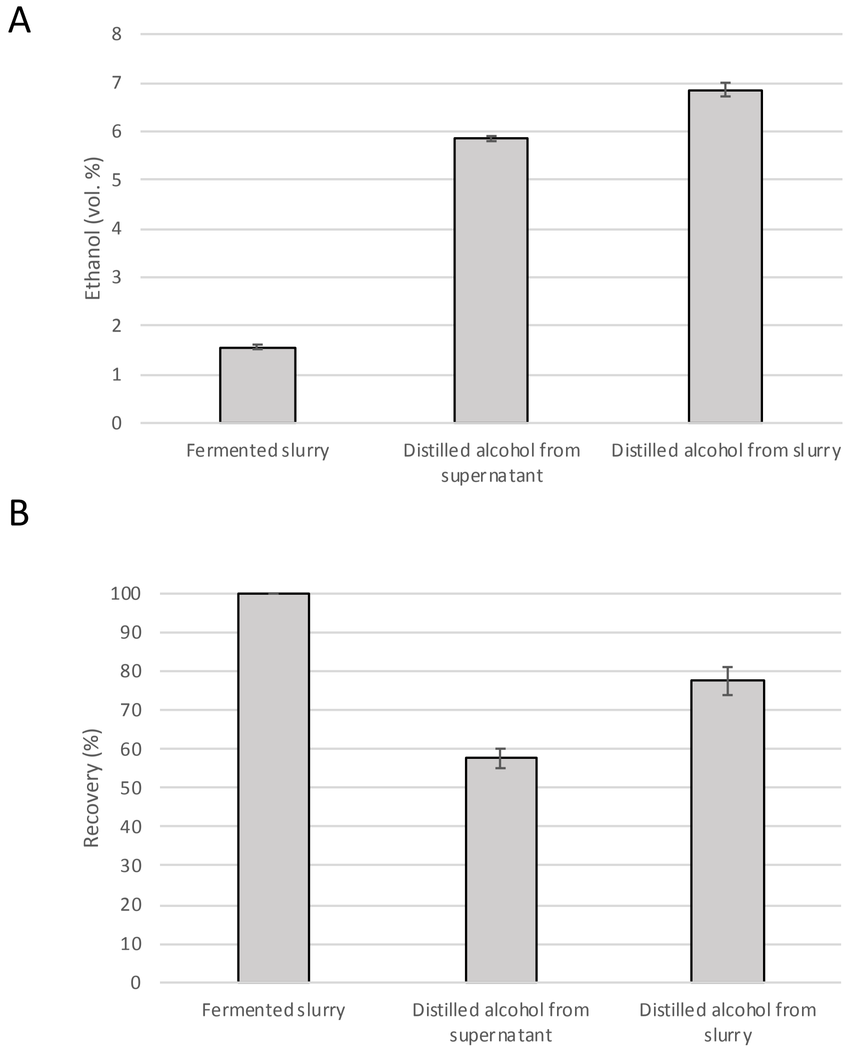

Fig.S2 Alcohol concentrations with distillation processes and recovery of alcohol from fermented slurry.

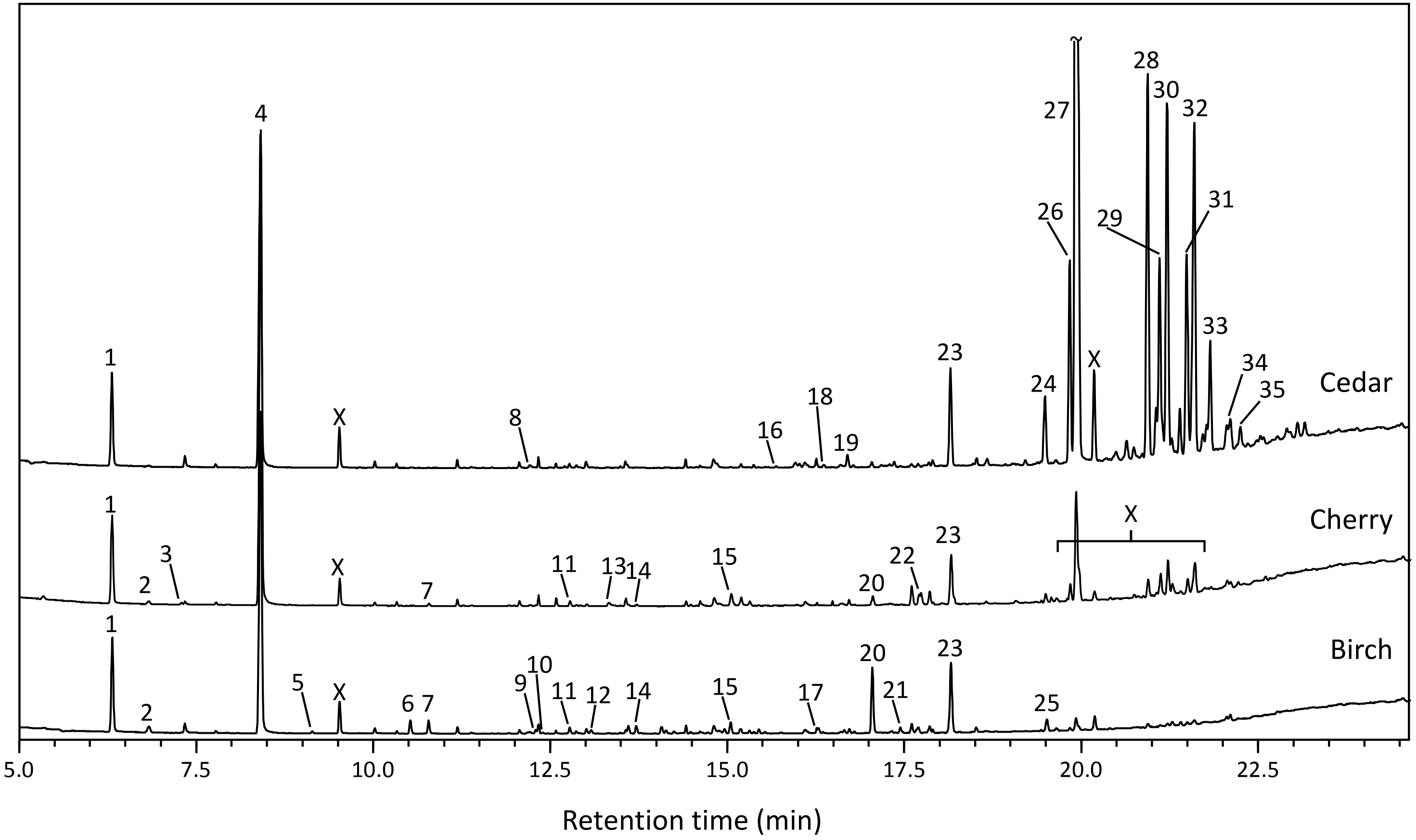

Fig. S3 Total ion-chromatograms of SPME-GC-MS analysis of Cedar, Cherry and Birch distillates.

Figure S3. *Otsuka et al.*
